# Supplementary material for: Seasonal and longitudinal water quality dynamics in three effluent-dependent rivers in Arizona
Source: PeerJ. 2023 Mar 29;11:e15069. doi: 10.7717/peerj.15069 (PMC10066693; doi:10.7717/peerj.15069)
Supplement: Supplemental Information 2 — With predictors distance from outfall in km (Distance), season (Spring, Summer, Winter), and the interaction between distance and season with river reach (Reach) as a random factor in the model. Results include Beta estimates (Estimates) with corresponding 95% confidence intervals (CI) and p values (p) as well as within-reach residual variance (σ2), between reach variance (τ00), and intra-class correlation coefficient (ICC) for the random factor. Both marginal and conditional pseudo R2 values are listed. Bold indicates significant p values of predictors (α ≤ 0.05). [file peerj-11-15069-s002.docx]

**Supplemental Materials**

**Table S1.** Summary Linear Mixed-Effect model results explaining variation in **alkalinity, conductivity, pH, nitrate, and phosphorous** (total phosphorous) with predictors distance from outfall in km (Distance), season (Spring, Summer, Winter), and the interaction between distance and season with river reach (Reach) as a random factor in the model. Results include Beta estimates (Estimates) with corresponding 95% confidence intervals (CI) and *p* values (*p*) as well as within-reach residual variance (σ^2^), between reach variance (τ_00_), and intra-class correlation coefficient (ICC) for the random factor. Both marginal and conditional pseudo R^2^ values are listed. Bold indicates significant *p* values of predictors (α ≤ 0.05).

|  | **Alkalinity** | | | **Conductivity** | | | **pH** | | |  | **Nitrate** |  | **Phosphorous** | | |
| --- | --- | --- | --- | --- | --- | --- | --- | --- | --- | --- | --- | --- | --- | --- | --- |
| **Predictors** | *Estimates* | *CI* | *p* | *Estimates* | *CI* | *p* | *Estimates* | *CI* | *p* | *Estimates* | *CI* | *p* | *Estimates* | *CI* | *p* |
| (Intercept) | 172.65 | 152.20 – 193.11 | **<0.001** | 1051.65 | 575.79– 1527.50 | **<0.001** | 8.04 | 7.63 – 8.45 | **<0.001** | 2.12 | 1.36 – 2.88 | **<0.001** | 1.28 | 0.85 – 1.71 | **<0.001** |
| Distance | 0.18 | -0.68 – 1.03 | 0.684 | 1.55 | -3.62 – 6.73 | 0.556 | 0.01 | -0.00 – 0.02 | 0.203 | 0.03 | -0.01 – 0.07 | 0.180 | -0.01 | -0.02 – 0.00 | 0.124 |
| Spring | 2.99 | -15.12 – 21.09 | 0.747 | 142.52 | 33.08– 251.96 | **0.011** | -0.12 | -0.41 – 0.17 | 0.420 | 0.21 | -0.61 – 1.04 | 0.612 | -0.02 | -0.28 – 0.24 | 0.854 |
| Summer | -19.90 | -37.75 – -2.05 | **0.029** | 100.72 | -7.17 – 208.61 | 0.067 | -0.80 | -1.09 – -0.52 | **<0.001** | 0.22 | -0.60 – 1.04 | 0.597 | 0.44 | 0.18 – 0.70 | **0.001** |
| Winter | -14.34 | -32.17 – 3.48 | 0.115 | 59.81 | -47.95 – 167.56 | 0.277 | 0.06 | -0.22 – 0.35 | 0.656 | 0.53 | -0.29 – 1.35 | 0.204 | 0.40 | 0.15 – 0.66 | **0.002** |
| Distance*Spring | -0.68 | -1.87 – 0.51 | 0.263 | -2.88 | -10.06 – 4.31 | 0.433 | 0.01 | -0.01 – 0.03 | 0.318 | 0.00 | -0.05 – 0.05 | 0.996 | 0.02 | -0.00 – 0.03 | 0.061 |
| Distance*Summer | 0.97 | -0.21 – 2.15 | 0.108 | -0.48 | -7.62– 6.65 | 0.894 | 0.04 | 0.02 – 0.06 | **<0.001** | -0.05 | -0.11 – 0.00 | 0.052 | -0.01 | -0.03 – 0.01 | 0.205 |
| Distance*Winter | 0.40 | -0.71 – 1.51 | 0.480 | 1.50 | -5.18 – 8.19 | 0.659 | 0.01 | -0.00 – 0.03 | 0.147 | 0.00 | -0.05 – 0.05 | 0.908 | -0.01 | -0.03 – 0.01 | 0.225 |
| Random Effects: |  |  |  |  |  |  |  |  |  |  |  |  |  |  |  |
| σ^2^ | 723.90 |  |  | 26445.38 |  |  | 0.19 |  |  | 1.52 |  |  | 0.15 |  |  |
| τ_00_ Reach | 400.00 |  |  | 344409.29 |  |  | 0.20 |  |  | 0.37 |  |  | 0.23 |  |  |
| ICC | 0.36 |  |  | 0.93 |  |  | 0.51 |  |  | 0.20 |  |  | 0.61 |  |  |
| N_Site_ | 6 |  |  | 6 |  |  | 6 |  |  | 6 |  |  | 6 |  |  |
| N | 170 |  |  | 170 |  |  | 170 |  |  | 170 |  |  | 170 |  |  |
| Marginal R^2^ | 0.050 |  |  | 0.006 |  |  | 0.244 |  |  | 0.097 |  |  | 0.093 |  |  |
| Conditional R^2^ | 0.388 |  |  | 0.929 |  |  | 0.633 |  |  | 0.274 |  |  | 0.644 |  |  |
