# Supplementary material for: Seasonal and longitudinal water quality dynamics in three effluent-dependent rivers in Arizona
Source: PeerJ. 2023 Mar 29;11:e15069. doi: 10.7717/peerj.15069 (PMC10066693; doi:10.7717/peerj.15069)
Supplement: Supplemental Information 3 [file peerj-11-15069-s003.docx]

**Supplemental Materials**

**Table S2.** Flow discharge volume, maximum and minimum air temperatures on all sampling dates for each site and reach across the study period.

| **Date** | **Sites** | **Flow Estimate (cfs)** | **Max. Air Temp.** | **Min. Air Temp.** |
| --- | --- | --- | --- | --- |
| **Santa Cruz River - Agua Nueva Reach** | | | | |
| 1-Feb-18 | CC Park | 18 | 27.8 | 7.2 |
|  | Sunset | 17 | 27.8 | 7.2 |
|  | West Belmont Rd. | 15 | 27.8 | 7.2 |
| 1-Jun-18 | CC Park | 18 | 37.2 | 18.9 |
|  | Sunset Rd | 12 | 37.2 | 18.9 |
|  | West Belmont Rd. | 5 | 37.2 | 18.9 |
| 1-Sep-18 | CC Park | 18 | 35 | 27.2 |
|  | Sunset Rd | 15 | 35 | 27.2 |
|  | West Belmont Rd. | 9 | 35 | 27.2 |
| 1-Nov-18 | CC Park | 18 | 22.2 | 8.3 |
|  | Sunset Rd | 16 | 22.2 | 8.3 |
|  | West Belmont Rd. | 8 | 22.2 | 8.3 |
| **Salt River - 91^st^ Ave. Reach** | | | | |
| 17-Feb-18 | Outfall WWTP | 170 | 21.1 | 10 |
|  | S 109 Ave | 155 | 21.1 | 10 |
|  | S El Mirage Rd. | 130 | 21.1 | 10 |
| 7-Jun-18 | Outfall WWTP | 140 | 39.4 | 25 |
|  | S 109 Ave | 115 | 39.4 | 25 |
|  | S El Mirage Rd. | 84 | 39.4 | 25 |
| 16-Sep-18 | Outfall WWTP | 150 | 39.4 | 30 |
|  | S 109 Ave | 115 | 39.4 | 30 |
|  | S El Mirage Rd. | 85 | 39.4 | 30 |
| 21-Nov-18 | Outfall WWTP | 150 | 23.9 | 11.1 |
|  | S 109 Ave | 120 | 23.9 | 11.1 |
|  | S El Mirage Rd. | 95 | 23.9 | 11.1 |
| **Rio de Flag River - Central Reach** | | | | |
| 1-Mar-18 | RDF1 | 2 | 4.4 | -8.9 |
|  | RDF2 | 1.8 | 4.4 | -8.9 |
|  | RDF3 | 1.6 | 4.4 | -8.9 |
| 19-Jun-18 | RDF1 | 1.4 | 27.8 | 6.1 |
|  | RDF2 | 1.1 | 27.8 | 6.1 |
|  | RDF3 | 0.7 | 27.8 | 6.1 |
| 23-Jul-18 | RDF1 | 1.5 | 31.1 | 13.3 |
|  | RDF2 | 1.3 | 31.1 | 13.3 |
|  | RDF3 | 1.0 | 31.1 | 13.3 |
| 5-Nov-18 | RDF1 | 0.8 | 15.6 | -1.1 |
|  | RDF2 | 0.7 | 15.6 | -1.1 |
|  | RDF3 | 0.5 | 15.6 | -1.1 |
| **Rio de Flag River – Wildcat Reach** | | | | |
| 1-Mar-18 | WH1 | 5.5 | 4.4 | -8.9 |
|  | WH2 | 3.7 | 4.4 | -8.9 |
|  | WH3 | 1.9 | 4.4 | -8.9 |
|  | WH4 | DRY | DRY | DRY |
| 18-Jun-18 | WH1 | 2.5 | 23.9 | 2.8 |
|  | WH2 | 2.1 | 23.9 | 2.8 |
|  | WH3 | 1.8 | 23.9 | 2.8 |
|  | WH4 | 0.9 | 23.9 | 2.8 |
| 18-Jul-18 | WH1 | 4.5 | 26.7 | 15 |
|  | WH2 | 3.6 | 26.7 | 15 |
|  | WH3 | 2.9 | 26.7 | 15 |
|  | WH4 | 1.8 | 26.7 | 15 |
| 5-Nov-18 | WH1 | 4.0 | 15.6 | -1.1 |
|  | WH2 | 3.7 | 15.6 | -1.1 |
|  | WH3 | 3.1 | 15.6 | -1.1 |
|  | WH4 | 2.2 | 15.6 | -1.1 |
| **Santa Cruz River - Tres Rios Reach** | | | | |
| 1-Jan-18 | Cortaro | 47 | 23.9 | 6.7 |
|  | Twin Peaks | 41 | 23.9 | 6.7 |
|  | Avra Valley | 35 | 23.9 | 6.7 |
|  | Tangerine | 27 | 23.9 | 6.7 |
|  | Sanders | 22 | 23.9 | 6.7 |
|  | Marana | 16 | 23.9 | 6.7 |
|  | Trico | 12.6 | 23.9 | 6.7 |
|  | Hardin | 8 | 23.9 | 6.7 |
| 1-Feb-18 | Cortaro | 42 | 25.6 | 6.7 |
|  | Twin Peaks | 37 | 25.6 | 6.7 |
|  | Avra Valley | 34 | 25.6 | 6.7 |
|  | Tangerine | 24 | 25.6 | 6.7 |
|  | Sanders | 15 | 25.6 | 6.7 |
|  | Marana | 9 | 25.6 | 6.7 |
|  | Trico | 6.7 | 25.6 | 6.7 |
|  | Hardin | 3 | 25.6 | 6.7 |
| 1-Mar-18 | Cortaro | 51 | 18.3 | 1.1 |
|  | Twin Peaks | 42 | 18.3 | 1.1 |
|  | Avra Valley | 34 | 18.3 | 1.1 |
|  | Tangerine | 24 | 18.3 | 1.1 |
|  | Sanders | 11 | 18.3 | 1.1 |
|  | Marana | 5 | 18.3 | 1.1 |
|  | Trico | 1 | 18.3 | 1.1 |
|  | Hardin | 0.3 | 18.3 | 1.1 |
| 1-Apr-18 | Cortaro | 56.7 | 31.1 | 17.8 |
|  | Twin Peaks | 47 | 31.1 | 17.8 |
|  | Avra Valley | 39 | 31.1 | 17.8 |
|  | Tangerine | 31 | 31.1 | 17.8 |
|  | Sanders | 10 | 31.1 | 17.8 |
|  | Marana | 2 | 31.1 | 17.8 |
|  | Trico | 0.3 | 31.1 | 17.8 |
|  | Hardin | DRY | DRY | DRY |
| 1-May-18 | Cortaro | 46.6 | 27.8 | 16.1 |
|  | Twin Peaks | 39 | 27.8 | 16.1 |
|  | Avra Valley | 34 | 27.8 | 16.1 |
|  | Tangerine | 27 | 27.8 | 16.1 |
|  | Sanders | 7 | 27.8 | 16.1 |
|  | Marana | 2 | 27.8 | 16.1 |
|  | Trico | 0.5 | 27.8 | 16.1 |
|  | Hardin | 0.1 | 27.8 | 16.1 |
| 1-Jun-18 | Cortaro | 42.8 | 35.6 | 17.2 |
|  | Twin Peaks | 37 | 35.6 | 17.2 |
|  | Avra Valley | 34 | 35.6 | 17.2 |
|  | Tangerine | 26 | 35.6 | 17.2 |
|  | Sanders | 12 | 35.6 | 17.2 |
|  | Marana | 7 | 35.6 | 17.2 |
|  | Trico | 4.2 | 35.6 | 17.2 |
|  | Hardin | DRY | DRY | DRY |
| 1-Jul-18 | Cortaro | 18.9 | 37.2 | 17.8 |
|  | Twin Peaks | 16 | 37.2 | 17.8 |
|  | Avra Valley | 14 | 37.2 | 17.8 |
|  | Tangerine | 11 | 37.2 | 17.8 |
|  | Sanders | 7 | 37.2 | 17.8 |
|  | Marana | 2 | 37.2 | 17.8 |
|  | Trico | 0.8 | 37.2 | 17.8 |
|  | Hardin | DRY | DRY | DRY |
| 1-Aug-18 | Cortaro | 49.2 | 41.1 | 26.1 |
|  | Twin Peaks | 41 | 41.1 | 26.1 |
|  | Avra Valley | 33 | 41.1 | 26.1 |
|  | Tangerine | 26 | 41.1 | 26.1 |
|  | Sanders | 9 | 41.1 | 26.1 |
|  | Marana | 3 | 41.1 | 26.1 |
|  | Trico | DRY | DRY | DRY |
|  | Hardin | DRY | DRY | DRY |
| 1-Sep-18 | Cortaro | 17.3 | 33.9 | 25 |
|  | Twin Peaks | 16 | 33.9 | 25 |
|  | Avra Valley | 14 | 33.9 | 25 |
|  | Tangerine | 12 | 33.9 | 25 |
|  | Sanders | 7 | 33.9 | 25 |
|  | Marana | 4 | 33.9 | 25 |
|  | Trico | 0.8 | 33.9 | 25 |
|  | Hardin | 0.1 | 33.9 | 25 |
| 13-Oct-18 | Cortaro Rd | 23.5 | 20 | 16.1 |
|  | Twin Peaks | 22 | 20 | 16.1 |
|  | Avra Valley | 20 | 20 | 16.1 |
|  | Tangerine | 17 | 20 | 16.1 |
|  | Sanders | 13 | 20 | 16.1 |
|  | Marana | 8 | 20 | 16.1 |
|  | Trico | 4.5 | 20 | 16.1 |
|  | Hardin | 2.8 | 20 | 16.1 |
| 7-Nov-18 | Cortaro Rd | 53.6 | 27.8 | 12.2 |
|  | Twin Peaks | 42 | 27.8 | 12.2 |
|  | Avra Valley | 34 | 27.8 | 12.2 |
|  | Tangerine | 27 | 27.8 | 12.2 |
|  | Sanders | 21 | 27.8 | 12.2 |
|  | Marana | 17 | 27.8 | 12.2 |
|  | Trico | 13 | 27.8 | 12.2 |
|  | Hardin | 8 | 27.8 | 12.2 |
| 19-Dec-18 | Cortaro Rd | 67.1 | 21.7 | 2.2 |
|  | Twin Peaks | 57 | 21.7 | 2.2 |
|  | Avra Valley | 49 | 21.7 | 2.2 |
|  | Tangerine | 38 | 21.7 | 2.2 |
|  | Sanders | 29 | 21.7 | 2.2 |
|  | Marana | 24 | 21.7 | 2.2 |
|  | Trico | 20.3 | 21.7 | 2.2 |
|  | Hardin | 15 | 21.7 | 2.2 |
| **Santa Cruz River – Nogales Reach** | | | | |
| 18-Jan-18 | Guy Tobin | 24 | 22.2 | 2.2 |
|  | Palo Parado | 21 | 22.2 | 2.2 |
|  | Santa Gertrudis | 19 | 22.2 | 2.2 |
|  | Tubac | 15.5 | 22.2 | 2.2 |
|  | Chavez Siding | 13 | 22.2 | 2.2 |
| 18-Mar-18 | Guy Tobin | 24 | 17.2 | 2.8 |
|  | Palo Parado | 22 | 17.2 | 2.8 |
|  | Santa Gertrudis | 20 | 17.2 | 2.8 |
|  | Tubac | 19.3 | 17.2 | 2.8 |
|  | Chavez Siding | 15 | 17.2 | 2.8 |
| 1-May-18 | Guy Tobin | 24 | 22.8 | 10.6 |
|  | Palo Parado | 16 | 22.8 | 10.6 |
|  | Santa Gertrudis | 9 | 22.8 | 10.6 |
|  | Tubac | 3.2 | 22.8 | 10.6 |
|  | Chavez Siding | DRY | DRY | DRY |
| 1-Jul-18 | Guy Tobin | 24 | 35 | 15.6 |
|  | Palo Parado | 15 | 35 | 15.6 |
|  | Santa Gertrudis | 7 | 35 | 15.6 |
|  | Tubac | 0.3 | 35 | 15.6 |
|  | Chavez Siding | 0.1 | 35 | 15.6 |
| 1-Sep-18 | Guy Tobin | 24 | 31.7 | 19.4 |
|  | Palo Parado | 19 | 31.7 | 19.4 |
|  | Santa Gertrudis | 14 | 31.7 | 19.4 |
|  | Tubac | 8 | 31.7 | 19.4 |
|  | Chavez Siding | DRY | DRY | DRY |
| 1-Nov-18 | Guy Tobin | 27 | 21.1 | 2.2 |
|  | Palo Parado | 25 | 21.1 | 2.2 |
|  | Santa Gertrudis | 24 | 21.1 | 2.2 |
|  | Tubac | 23.9 | 21.1 | 2.2 |
|  | Chavez Siding | 20 | 21.1 | 2.2 |
