# Supplementary material for: Seasonal and longitudinal water quality dynamics in three effluent-dependent rivers in Arizona
Source: PeerJ. 2023 Mar 29;11:e15069. doi: 10.7717/peerj.15069 (PMC10066693; doi:10.7717/peerj.15069)
Supplement: Supplemental Information 4 [file peerj-11-15069-s004.docx]

**Supplemental Materials**

**Table S3.** Average, minimum, and maximum water quality factor values across all sites by season.

| **Parameter** | **DO** | **Temperature** | **pH** | **Conductivity** | **Salinity** | **Alkalinity** | **Tot. Phosphorus** | **Ammonia** | **Nitrate** |
| --- | --- | --- | --- | --- | --- | --- | --- | --- | --- |
| Unit | (mg/l) | (°C) |  | μS | ppt | (mg/l CaCO_3_) | (mg/l P) | (mg/l N) | (mg/l N) |
| **Winter** | | | | | | | | | |
| Average | 8.4 | 19.2 | 8.4 | 1088.0 | 0.5 | 158.4 | 1.5 | 0.2 | 3.0 |
| Minimum | 5.4 | 14.1 | 7.4 | 620.0 | 0.3 | 108.3 | 0.2 | 0.0 | 1.2 |
| Maximum | 15.6 | 25.0 | 9.7 | 2670.0 | 1.1 | 231.7 | 2.4 | 1.2 | 6.4 |
| **Spring** | | | | | | | | | |
| Average | 7.3 | 27.2 | 8.1 | 1160.8 | 0.6 | 160.6 | 1.4 | 0.2 | 2.4 |
| Minimum | 2.7 | 19.8 | 7.6 | 620.0 | 0.3 | 120.0 | 0.1 | 0.0 | 0.4 |
| Maximum | 14.3 | 34.0 | 9.3 | 3280.0 | 1.6 | 383.3 | 2.3 | 0.5 | 6.5 |
| **Summer** | | | | | | | | | |
| Average | 5.9 | 29.1 | 7.7 | 1098.0 | 0.5 | 158.1 | 1.6 | 0.7 | 2.0 |
| Minimum | 2.9 | 20.3 | 5.0 | 359.0 | 0.2 | 91.7 | 0.2 | 0.0 | 0.5 |
| Maximum | 9.0 | 34.2 | 8.7 | 3410.0 | 1.6 | 253.3 | 3.5 | 3.7 | 5.8 |
| **Fall** | | | | | | | | | |
| Average | 9.3 | 20.1 | 8.1 | 1043.5 | 0.5 | 165.4 | 1.3 | 0.9 | 2.3 |
| Minimum | 4.8 | 9.2 | 7.5 | 635.0 | 0.2 | 75.0 | 0.4 | 0.0 | 0.1 |
| Maximum | 20.6 | 28.5 | 9.9 | 2690.0 | 1.2 | 215.0 | 2.1 | 5.4 | 6.4 |
