# Supplementary material for: Seasonal and longitudinal water quality dynamics in three effluent-dependent rivers in Arizona
Source: PeerJ. 2023 Mar 29;11:e15069. doi: 10.7717/peerj.15069 (PMC10066693; doi:10.7717/peerj.15069)
Supplement: Supplemental Information 5 [file peerj-11-15069-s005.pdf]

Supplemental Figure

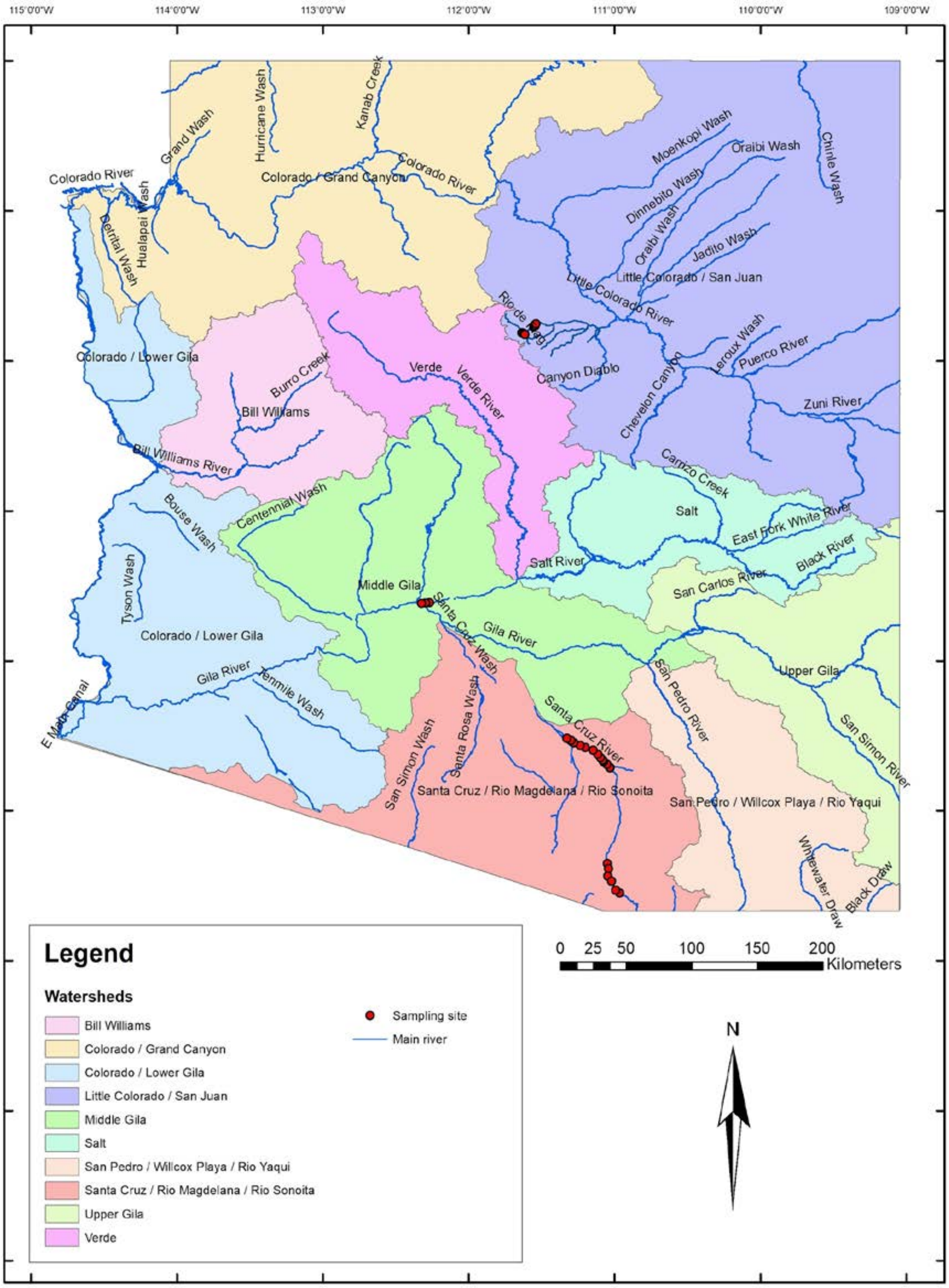

Figure S1. Locations of all sampling sites within each of the six study reaches across the three focal river basins in Arizona
