# Supplementary material for: Seasonal and longitudinal water quality dynamics in three effluent-dependent rivers in Arizona
Source: PeerJ. 2023 Mar 29;11:e15069. doi: 10.7717/peerj.15069 (PMC10066693; doi:10.7717/peerj.15069)
Supplement: Supplemental Information 6 [file peerj-11-15069-s006.pdf]

Supplemental Figures

Dissolved Oxygen

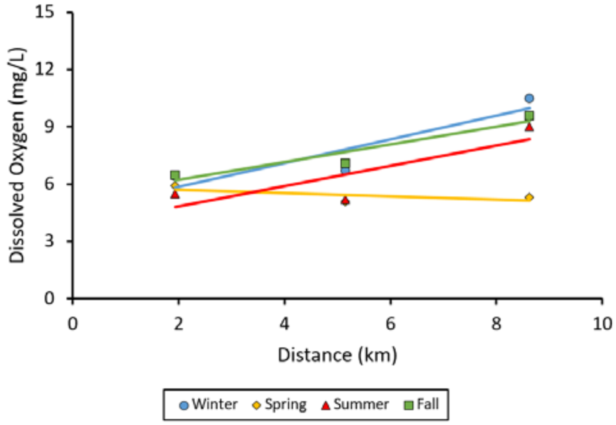

Figure S2a. Dissolved oxygen at the Agua Nueva reach of the Santa Cruz River, Arizona.

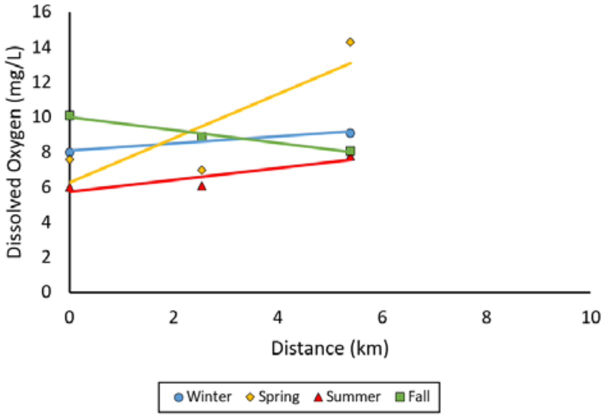

Figure S2b. Dissolved oxygen at the 91st Ave reach of the Salt River, Arizona.

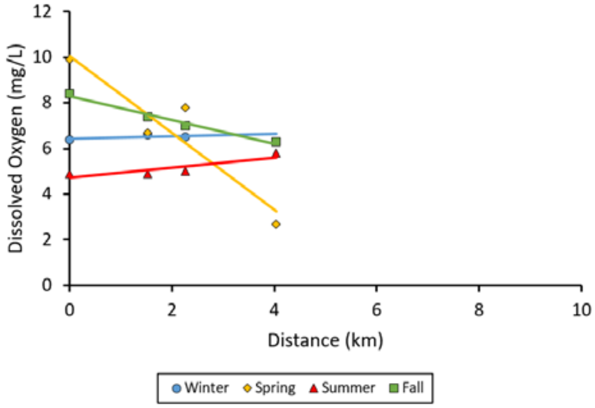

Figure S2c. Dissolved Oxygen at the Wildcat reach of the Rio de Flag, Arizona.

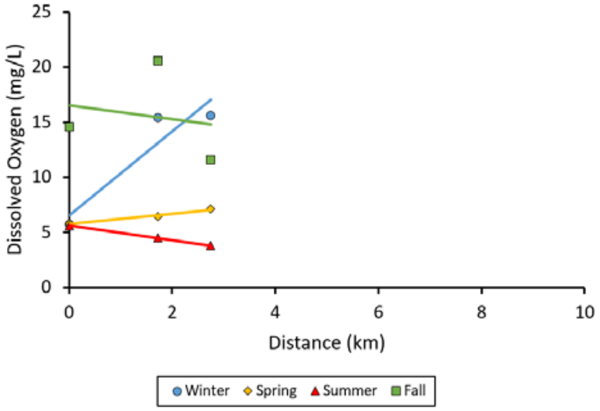

Figure S2d. Dissolved Oxygen at the Central reach of the Rio de Flag, Arizona.

Temperature

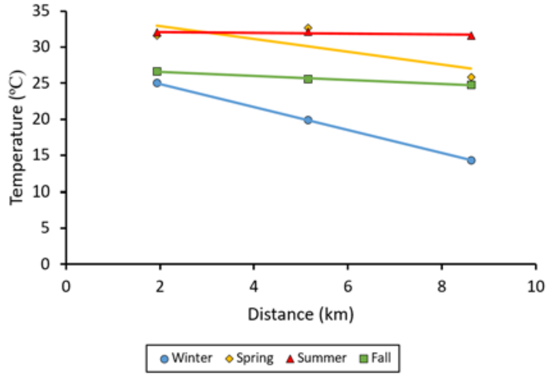

Figure S2e. Temperature at the Agua Nueva reach of the Santa Cruz River, Arizona.

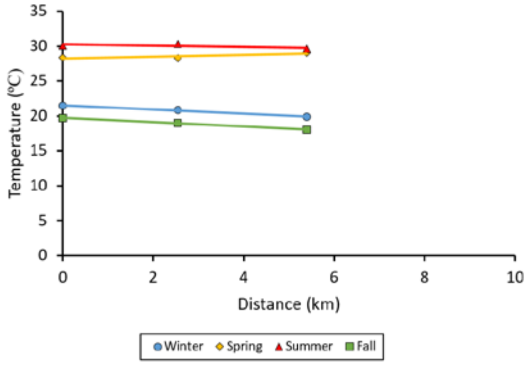

Figure S2f. Temperature at the 91st Ave reach of the Salt River, Arizona.

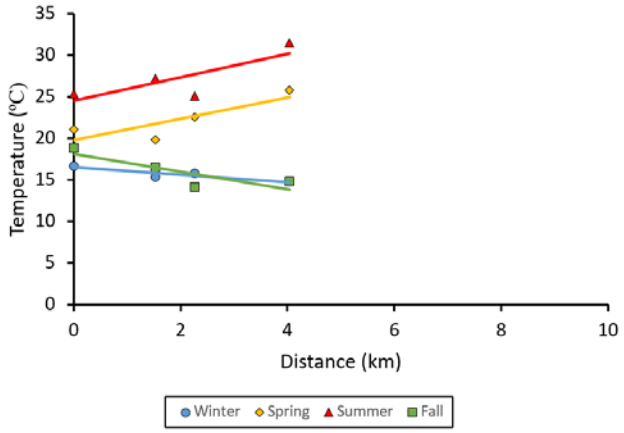

Figure S2g. Temperature at the Wildcat reach of the Rio de Flag, Arizona.

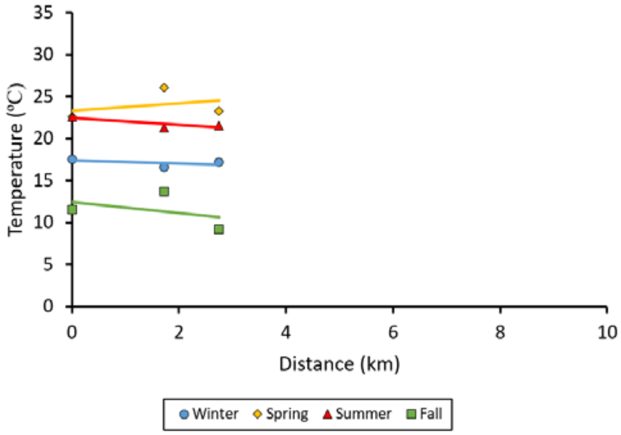

Figure S2h. Temperature at the Central reach of the Rio de Flag, Arizona.

pH

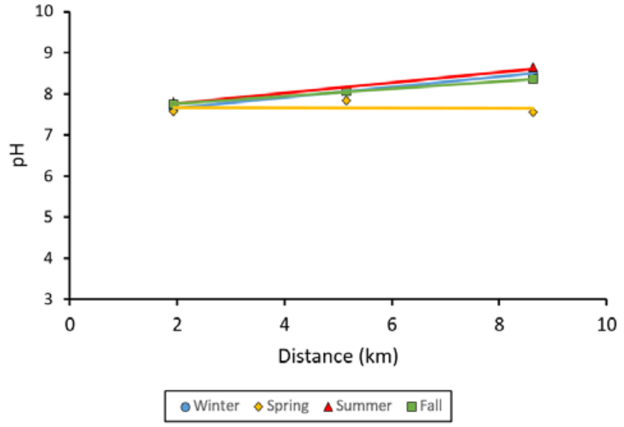

Figure S2i. pH at the Agua Nueva reach of the Santa Cruz River, Arizona.

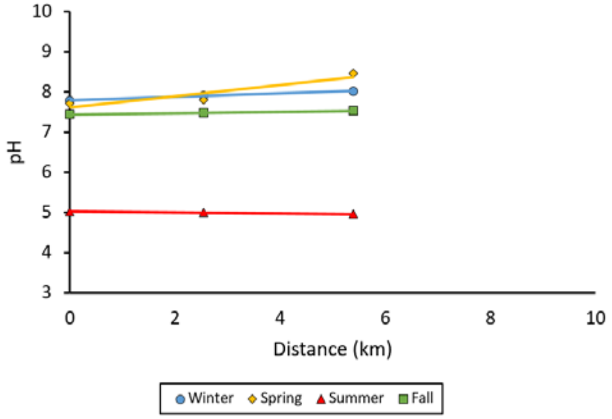

Figure S2j. pH at the 91st Ave reach of the Salt River, Arizona.

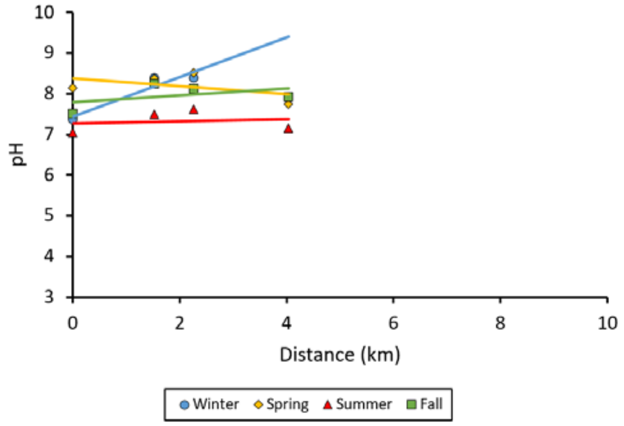

Figure S2k. pH at the Wildcat reach of the Rio de Flag, Arizona.

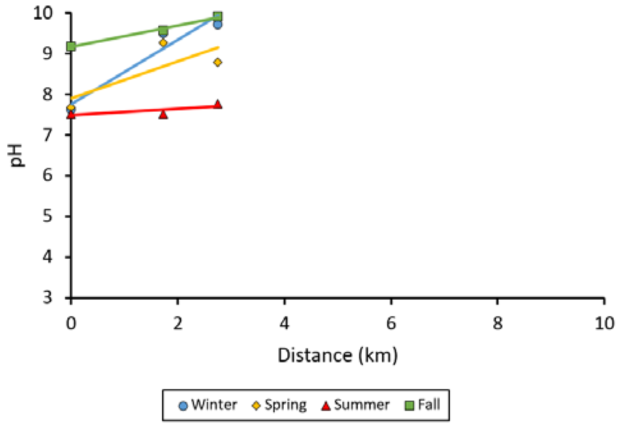

Figure S2l. pH at the Central reach of the Rio de Flag, Arizona.

Conductivity

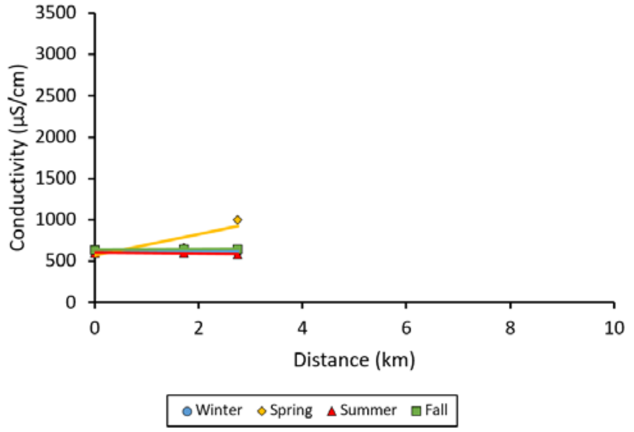

Figure S2m. Conductivity at the Central reach of the Rio de Flag, Arizona.

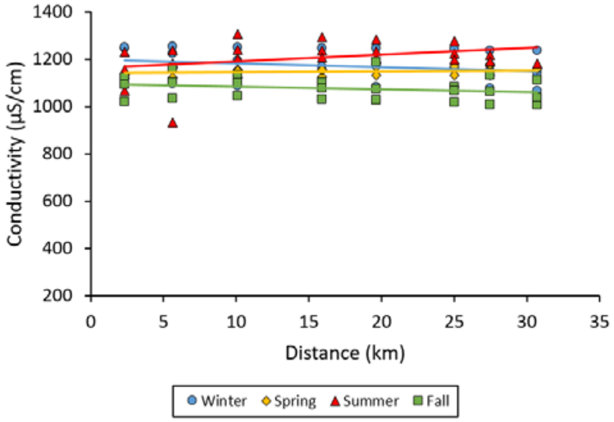

Figure S2n. Conductivity at the Tres Rios reach of the Santa Cruz River, Arizona.

Alkalinity

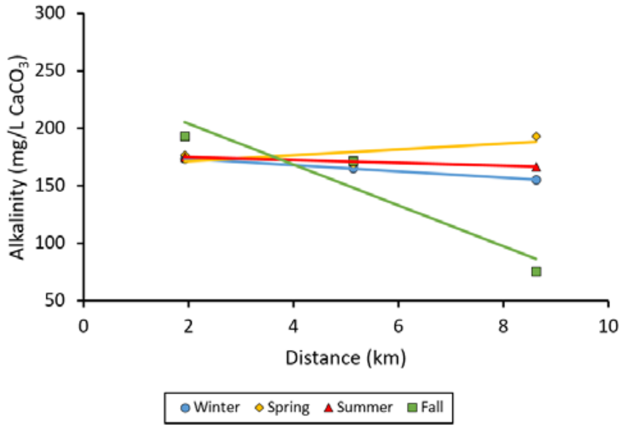

Figure S2o. Alkalinity at the Agua Nueva reach of the Santa Cruz River, Arizona.

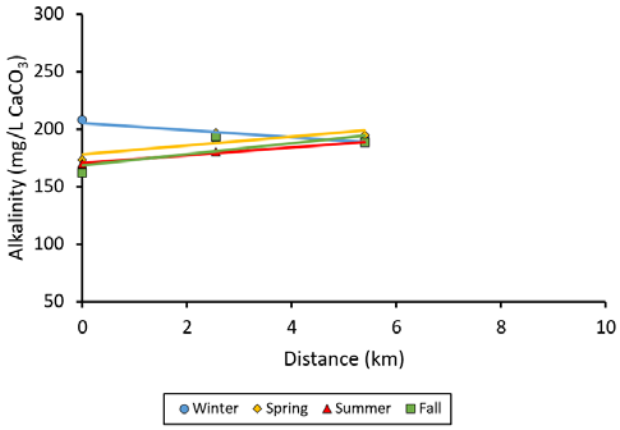

Figure S2p. Alkalinity at the 91st Ave reach of the Salt River, Arizona.

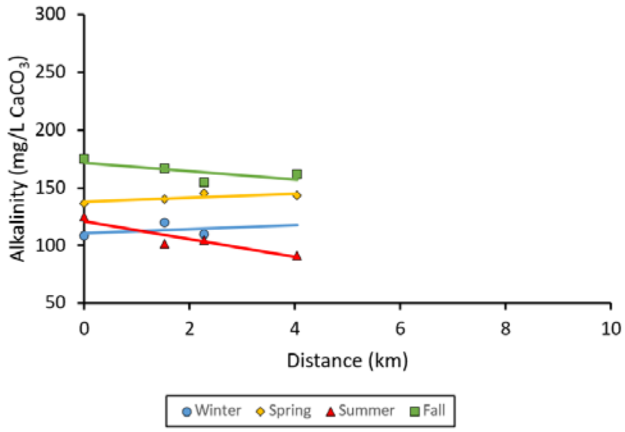

Figure S2q. Alkalinity at the Wildcat reach of the Rio de Flag, Arizona.

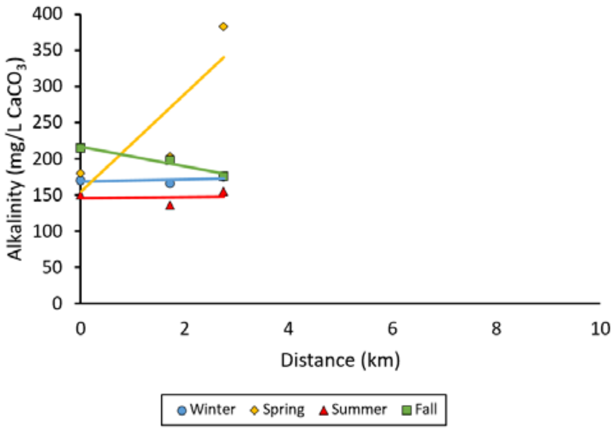

Figure S2r. Alkalinity at the Central reach of the Rio de Flag, Arizona.

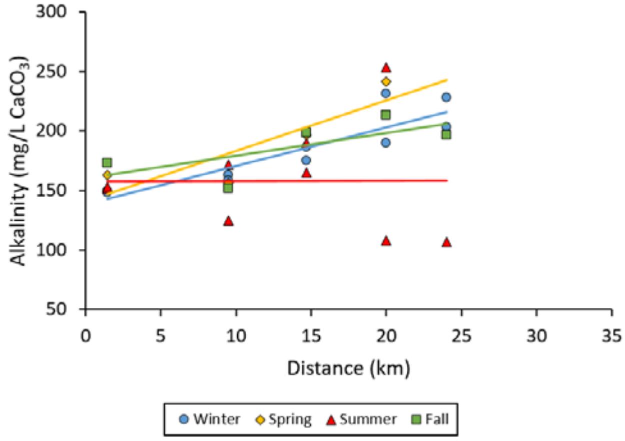

Figure S2s. Alkalinity at the Nogales reach of the Santa Cruz River, Arizona.

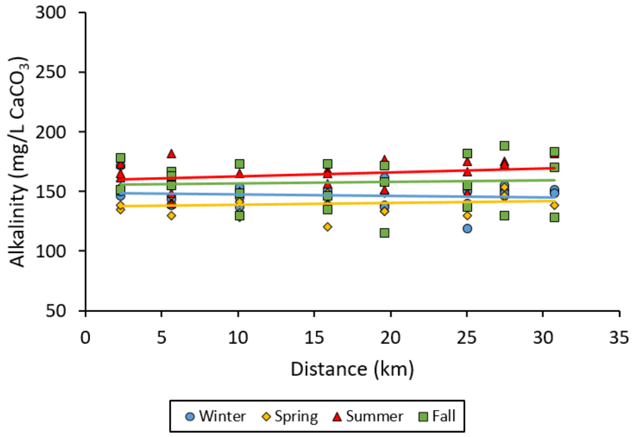

Figure S2t. Alkalinity at the Tres Rios reach of the Santa Cruz River, Arizona.

Phosphorus

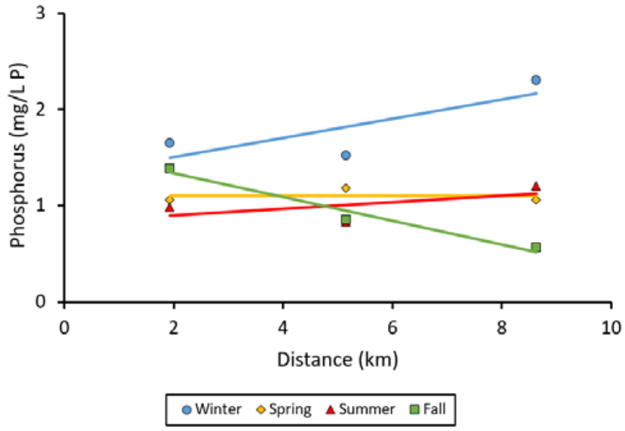

Figure S2u. Phosphorus at the Agua Nueva reach of the Santa Cruz River, Arizona.

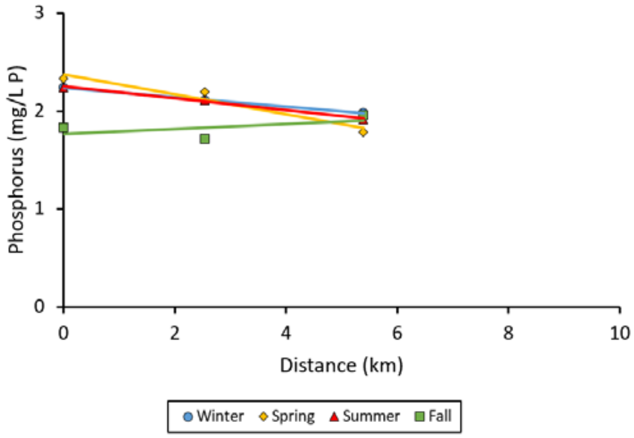

Figure S2v. Phosphorus at the 91st Ave reach of the Salt River, Arizona.

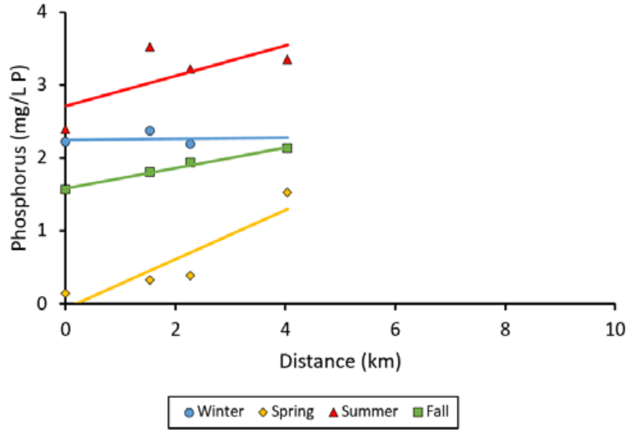

Figure S2w. Phosphorus at the Wildcat reach of the Rio de Flag, Arizona.

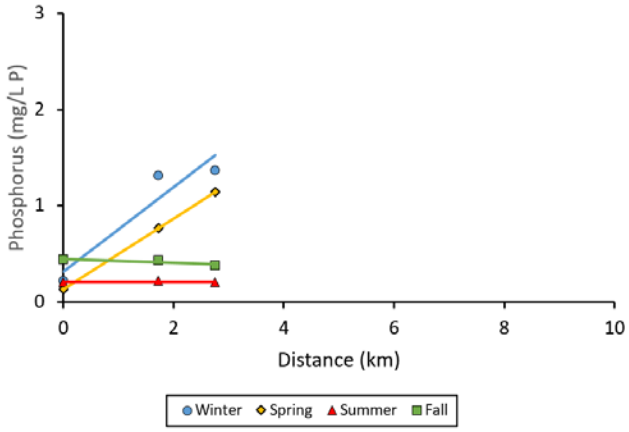

Figure S2x. Phosphorus at the Central reach of the Rio de Flag, Arizona.

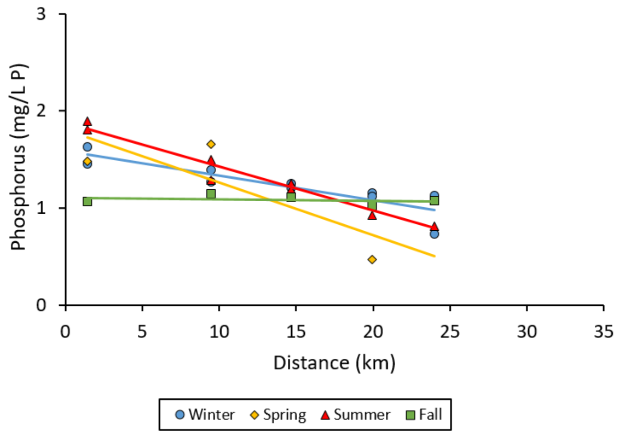

Figure S2y. Phosphorus at the Nogales reach of the Santa Cruz River, Arizona.

Ammonia

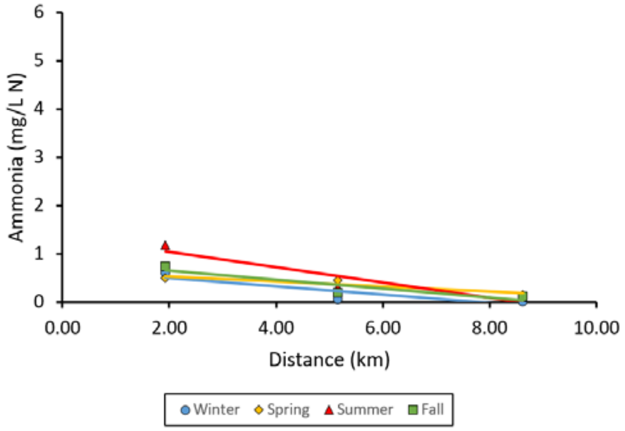

Figure S2z. Ammonia at the Agua Nueva reach of the Santa Cruz River, Arizona.

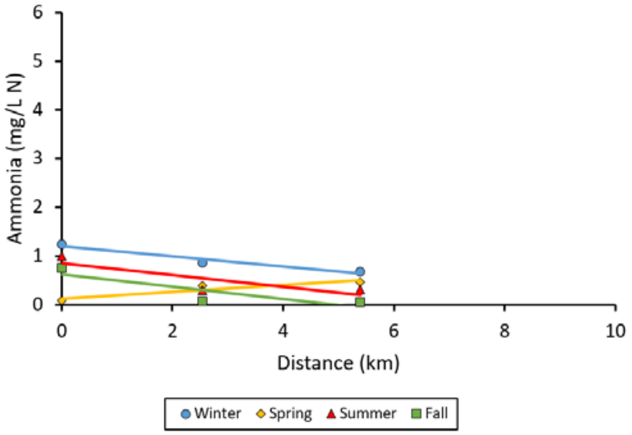

Figure S2aa. Ammonia at the 91st Ave reach of the Salt River, Arizona.

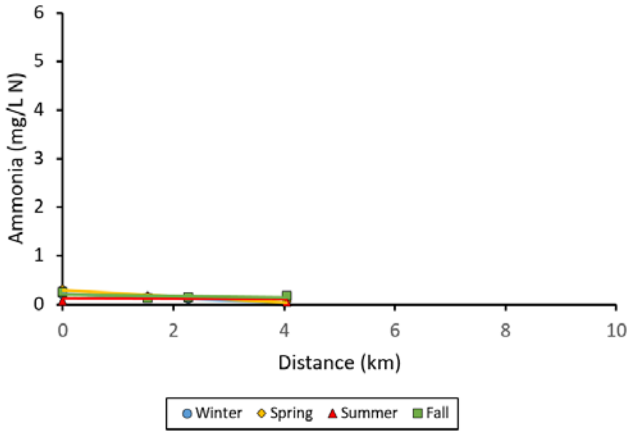

Figure S2ab. Ammonia at the Wildcat reach of the Rio de Flag, Arizona.

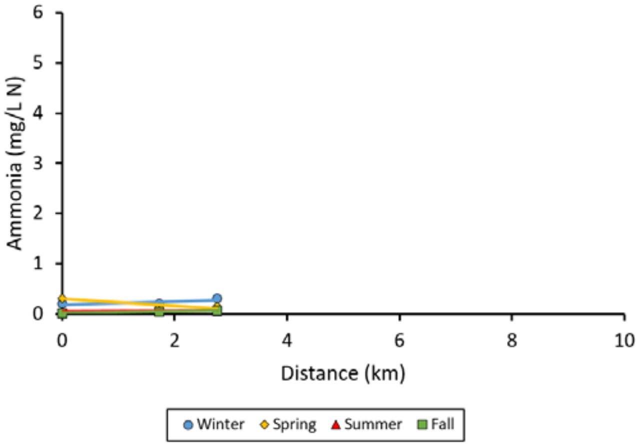

Figure S2ac. Ammonia at the Central reach of the Rio de Flag, Arizona.

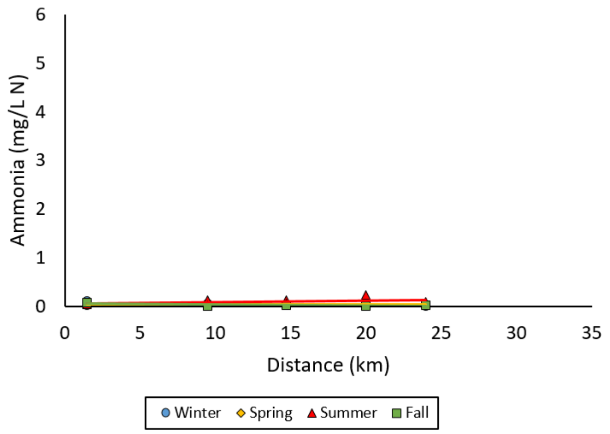

Figure S2ad. Ammonia at the Nogales reach of the Santa Cruz River, Arizona.

Nitrate

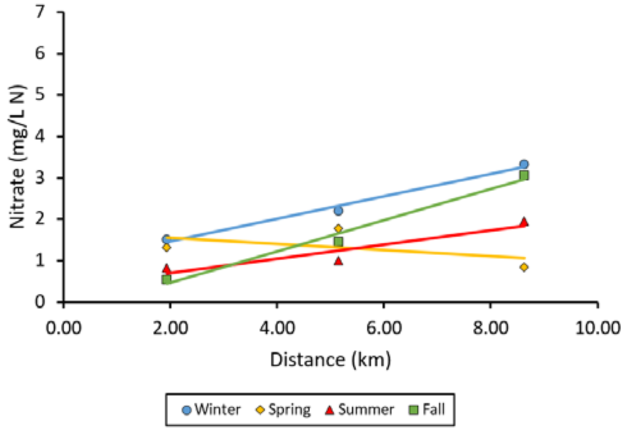

Figure S2ae. Nitrate at the Agua Nueva reach of the Santa Cruz River, Arizona.

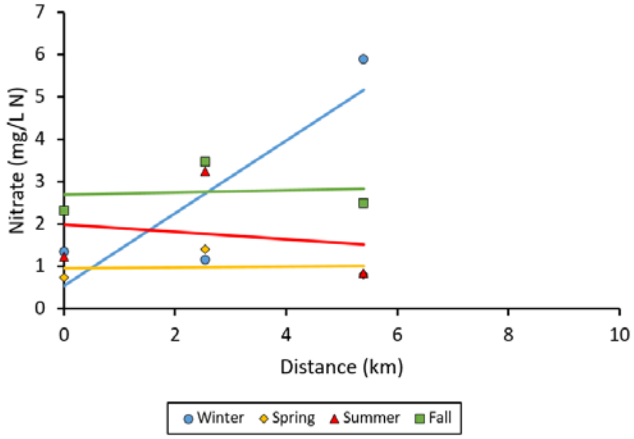

Figure S2af. Nitrate at the 91st Ave reach of the Salt River, Arizona.

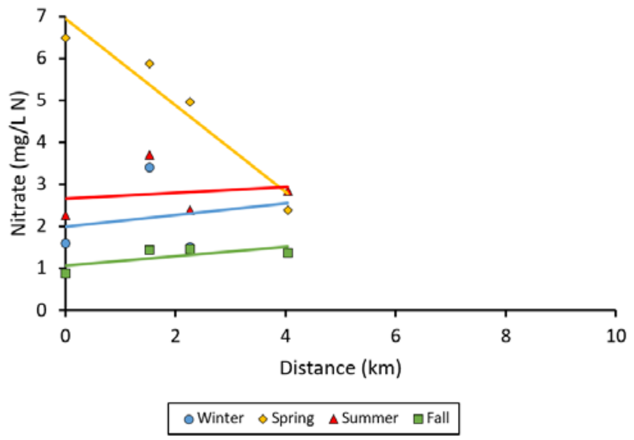

Figure S2ag. Nitrate at the Wildcat reach of the Rio de Flag, Arizona.

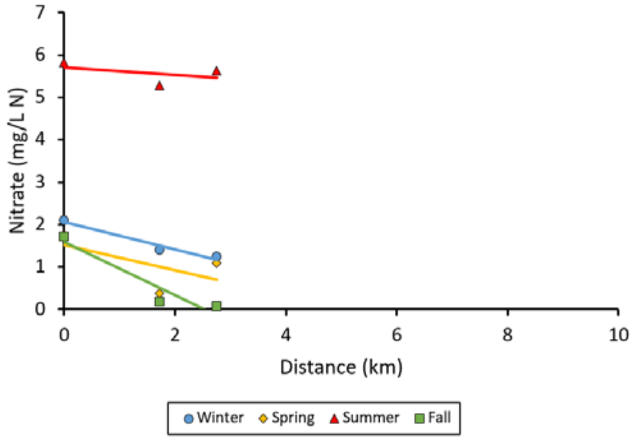

Figure S2ah. Nitrate at the Central reach of the Rio de Flag, Arizona.

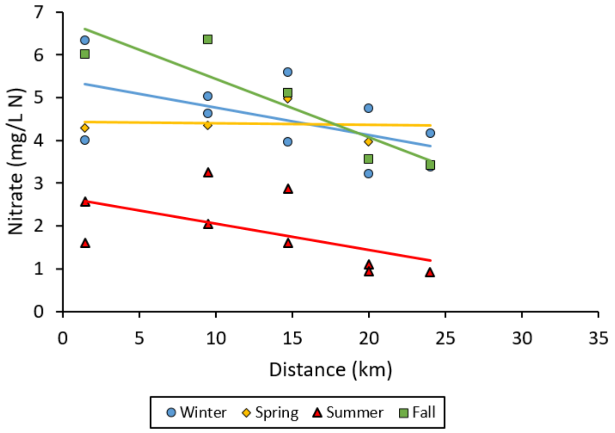

Figure S2ai. Nitrate at the Nogales reach of the Santa Cruz River, Arizona.
